# Decreased eating frequency linked to increased visceral adipose tissue, body fat, and BMI in Hispanic college freshmen

**DOI:** 10.1186/s40795-018-0217-z

**Published:** 2018-03-06

**Authors:** Benjamin T. House, Grace E. Shearrer, Jessica B. Boisseau, Molly S. Bray, Jaimie N. Davis

**Affiliations:** Department of Nutritional Sciences, University of Texas (BTH, GES, JBB, MSB, JND), 103 W 24th St, Austin, TX 78712 USA

**Keywords:** Eating frequency, Visceral fat, Adiposity, College, Hispanic

## Abstract

**Background:**

To investigate the relationship between eating frequency and specific adiposity markers in a potentially high-risk and understudied population of Hispanic college freshmen.

**Methods:**

This study included 92 Hispanic college freshmen (18–19 y). The following cross-sectional data were collected: height, weight, waist circumference, body mass index (BMI), dietary intake, body composition, physical activity, hepatic fat, visceral adipose tissue (VAT), and subcutaneous adipose tissue (SAT).

**Results:**

Infrequent eaters ate 44% less often (2.5 ± 0.2 vs. 4.5 ± 0.8, *p* ≤ 0.01) and consumed 27% more calories per EO (*p* ≤ 0.01), while consuming 21% less kcals per day (*p* ≤ 0.01) compared to frequent eaters. Infrequent eaters had 8% higher BMIs (24.8 ± 4.4 vs. 22.9 ± 3.2 kg/m^2^) (*p* = 0.02)*,* 60% higher BMI z-scores (0.5 ± 1.0 vs. 0.2 ± 1.0, *p* = 0.03)*,* 21% higher VAT (298.3 ± 153.8 vs. 236.8 ± 78.2 ml, *p* = 0.03), 26% higher SAT (1150.1 ± 765.4 vs. 855.6 ± 494.6 ml, *p* = 0.03), and 8% higher total body fat (27.6 ± 10.8 vs. 25.3 ± 8.8%, *p* = 0.04) compared to frequent eaters while showing no significant difference in physical activity. These findings seem to be driven by females more than males.

**Conclusions:**

These findings suggest that infrequent eating is related to increased adiposity in Hispanic college freshmen, despite a decreased daily energy intake and no significant differences in physical activity. Yet, more research is needed to understand the underlying mechanisms of these findings, as well as investigate any potential causal relationship between eating frequency and adiposity in Hispanic youth.

## Background

College students are particularly susceptible to poor overall health and the transition to college has been identified as a critical period contributing to the rise in obesity rates as the behavioral choices college students make likely affect their risk of chronic disease later in life [1]. In 2012, for the first time in US history, Hispanic high school graduates (69%) were more likely to be enrolled in college than Non-Hispanic Whites (NHW; 67%) and Blacks (63%) [2]. Hispanic students consistently represent around one quarter of freshman enrollment at the University of Texas at Austin [3]. Currently, 67% of Hispanics (12–19 y) are either overweight or obese [4], yet little is known about overweight or obese prevalence rates of Hispanic college students. Several studies have shown that the initial transition to college is associated with rapid weight gain and the average weight gain in the first year of college ranges from 3.5 to 8.8 pounds [5]. Decreased dietary fiber, fruits and vegetables [6, 7] and increased junk food consumption [8] are among the dietary factors linked to increased obesity rates in primarily NHW college students. However, no study has investigated the relationship eating frequency patterns and obesity risk in a population of exclusively Hispanic college freshmen nor has any study examined the potential sex differences in this relationship.

The Gibney et al. eating occasion definition was used to quantify eating frequency and each EO was defined as ≥50 cal and ≥15 min from any previous EO [9]. This definition was selected because it has been cited within the eating frequency literature most often [10–14], thus allowing us to compare our results with previous eating frequency research. Frequent eating was classified as averaging more than 4 EOs per day, while infrequent eating was classified as averaging less than 3 EOs per day.

To date, the majority of epidemiology studies have shown an inverse association between eating frequency and adiposity [13–19], while a few studies have found no relationship or a positive association [20–22]. Previous research has shown that infrequent eating is linked to increased visceral adipose tissue (VAT) and obesity risk, as well as blunted insulin action, and deleterious lipid parameters in multiple populations of overweight Hispanic youth (8–18 y) [13, 14]. Among minorities, Hispanics tend to have high amounts of VAT, and VAT is a strong indicator of deleterious metabolic profiles, such as dyslipidemia and glucose intolerance [23]. High amounts of VAT have also been related to Non-Alcoholic Fatty Liver Disease (NAFLD), which is also increasing within Hispanic youth populations [24]. Thus, examining the impact of eating patterns on specific fat depots in high-risk populations is warranted.

To date, no group has examined the effect of eating frequency on adiposity and metabolic disease risk in a sample of Hispanic college freshmen. Thus, the goal of this study is to examine the relationship between eating frequency and specific adiposity markers in this potentially high-risk and understudied population of Hispanic college freshmen to better inform interventions that may reduce this risk within such a crucial period of life. We hypothesized that infrequent eating in relation to frequent eating would be inversely associated with energy intake and physical activity, but positively associated with adiposity measures in Hispanic college freshmen and that these findings would be similar in both males and females.

## Methods

### Subjects

Inclusion criteria for the study were as follows: (i) self-reported that all four of their grandparents were of Hispanic origin (ii) 18–19 years of age, and (iii) in their first year of college. Exclusion criteria for the study were as follows: (i) pregnancy, (ii) taking any medication known to affect body composition or any psychoactive medication, (iii) diagnosed with a disease/s or syndrome known to affect body composition or fat distribution, (iv) a learning impairment that would complicate survey administration, (v) had braces, a pacemaker, or any of other contraindications to magnetic resonance imaging (MRI) scanning, or (vi) had taken part in a weight loss, dietary, or physical intervention in the previous 6 months. This study was approved by the Institutional Review Board. Informed written consent was obtained before testing commenced.

**Fig. 1:**
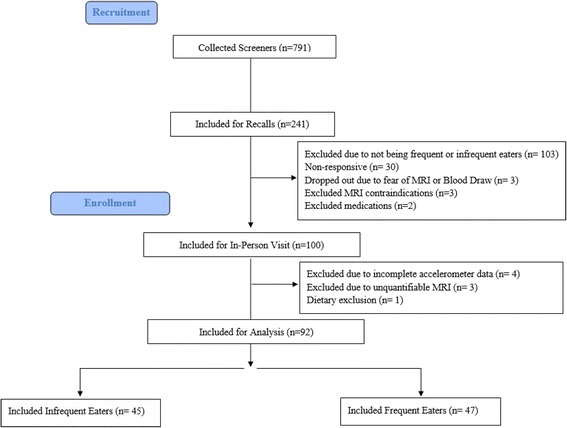
provides a detailed diagram of the inclusion and exclusion of study participants. 791 participants were recruited initially via a 21 item dietary screener, which asked about eating frequency habits and was adapted from Project Eat [25]. Only subjects who identified themselves as frequent or infrequent eaters on the survey were contacted for dietary recalls which were conducted by phone prior to the in-person data collection. Three or more 24 h multiple-pass dietary recalls were collected in 30 % (*n* = 241) of the total subject pool to verify eating frequency. Of those subjects, 43% (*n* = 103) were not frequent or infrequent eaters as determined via dietary recalls, and an additional 38 participants were either non-responsive or did not qualify due to other exclusionary criteria. Furthermore, only infrequent eaters who averaged less than 3 eating occasions (EOs) per 24 h or frequent eaters who averaged 4 or more EOs per 24 h on the majority of their dietary recalls were brought in for the in-person visit. There were a total of 100 subjects who completed the in-person visit, one subject did not have adequate dietary data, three participants did not have specific fat distribution data, and four subjects did not attain three days of eight hours or more of physical activity data via accelerometer, leaving the final sample size at 92 subjects

Figure 1 provides a detailed diagram of the inclusion and exclusion of study participants. Participants (*n* = 791) were recruited via announcement in classes, word of mouth, and electronic posted notices. Subjects initially completed a 21 item dietary screener, which asked about eating frequency habits and was adapted from Project Eat [25–27]. Only subjects who identified themselves as frequent or infrequent eaters on the survey were contacted for dietary recalls which were conducted by phone prior to in-person data collection. Furthermore, only infrequent eaters who averaged less than 3 eating occasions (EOs) per 24 h (*n* = 45) or frequent eaters who averaged 4 or more EOs per 24 h on the majority of their dietary recalls (*n* = 47) were brought in for the in-person visit. Three or more 24 h multiple-pass dietary recalls were collected in 30 % (*n* = 241) of the total subject pool to verify eating frequency. Of those subjects, 43% (*n* = 103) were not frequent or infrequent eaters as determined via dietary recalls, and an additional 38 participants were either non-responsive or did not qualify due to other exclusionary criteria. One-hundred subjects were then brought in for the in-person visit. There were a total of 100 subjects who completed the in-person visit, one subject did not have adequate dietary data, three participants did not have specific fat distribution data, and four subjects did not attain three days of eight hours or more of physical activity data via accelerometer, leaving the final sample size at 92 subjects.

### Anthropometrics and adiposity measures

Subjects arrived at approximately 0700 after an overnight fast, nothing to eat or drink except water after 2000 the night before. Height and weight were measured to the nearest 0.1 kg and 0.1 cm using a beam medical scale and a wall-mounted stadiometer, respectively, and the average of two measurements was used for the analysis. BMI percentiles and z-scores were determined by using EPII 2000 software (version 1.1; Centers for Disease Control and Prevention, Atlanta, GA) [28]. Subjects were categorized as overweight if they had a BMI of 25.0 to < 30.0 kg/m^2^ and obese if they had a BMI > 30.0 kg/m^2^ utilizing adult cut offs. Waist circumference was measured and recorded to the nearest 0.1 cm. Body fat and soft lean tissue were measured using air displacement plethysmography, which has been validated against hydrodensitometry in overweight adults (BOD POD, Cosmed 2007B, Concord, CA) [29].

### Image acquisition

Visceral adipose tissue (VAT), subcutaneous adipose tissue (SAT), and hepatic fat were assessed via magnetic resonance imaging (MRI) on a research-dedicated Siemens Skyra 3 Tesla scanner. Non-Alcoholic Fatty Liver Disease was categorized utilizing the percent fat cut point (more than 5.56%) derived from the Dallas Heart Study [30]. Visceral adipose tissue (VAT), subcutaneous adipose tissue (SAT), and liver fat volume were measured using a vibe 3D DIXON technique. This scanning protocol contained one slab with 88 slices, each 3 mm thick to allow for imaging of the entire abdominal area in the coronal direction. The field of view (FoV) was 380 mm, and the phase FoV was 80.6%, with a repetition time (TR) of 3.90 s, an echo time (TE) of 2.46 ms, and a flip angle of 9.0 degrees. One set each of in-phase images and one out-of-phase images were acquired. The Siemens Skyra 3 T used a portion of a 32-channel coil array integrated into the patient bed/Table. A 4-channel large array coil or a 4-channel small array coil was placed anteriorly and used in combination with the 32-channel coil to provide full abdominal coverage. Using a high number of coil elements in this way made an ideal experimental configuration to take advantage of a partially parallel image acquisition acceleration method. The GeneRalized Auto-calibrating Partially Parallel Acquisitions (GRAPPA) technique used for this study to accelerate image acquisition. Fat volume fraction and fat mass fraction were computed on a voxel-by-voxel basis, and averaged over each segmented organ. VAT and SAT were measured as a region of interest (ROI) from the top of the ileac crest to the bottom of the ribcage. The benefits of using ROI for liver fat and body fat quantification as well as challenges associated with fat quantification from MRI can be found here [29].

### Fat mass quantification

Percent water and fat for VAT and SAT were calculated using a novel quantification program developed by Dr. Jeff Luci and the Imaging Research Center staff based on the Otsu method and run in MATLAB (version R2013a, MathWorks Inc., Natick, MA). Initially, the total body fat was calculated and the subcutaneous fat was then calculated. The removal of the SAT from the total body fat yielded VAT. An average of 26 slices were taken from the abdominal area and there was not significant difference in the number of slices between groups. At least two researchers quantified the fat values for each subject. No significant differences in any of the outcome variables or MRI slices were seen between coders utilizing t-tests.

### Dietary intakes

Dietary intakes were assessed from three to four 24-h diet recalls using the multiple-pass technique. Research staff were trained and supervised by a Registered Dietitian. All subjects had at least three recalls (one weekend and two weekdays). On average recalls were administered within 5 days from the in-person testing visit. All dietary recall data was checked for errors in entry by additional trained research staff. Nutritional data was analyzed by using the Nutrition Data System for Research (NDS-R, 2014). The NDS-R program calculated key dietary variables for this analysis, including mean energy, total fat, protein, carbohydrates, saturated fat, total sugar, added sugar, dietary fiber, soluble fiber, and insoluble fiber. Prospectively, no recall was performed if the subject indicated being ill. One subject was excluded from all the analyses due to having a very low carbohydrate intake (< 10 g per day) and being an extreme outlier in percentage of caloric intake from carbohydrate. The dietary data was then examined for plausibility of energy intake according to the Willett exclusion criteria, but no male or female met the criteria for exclusion [31]. We further screened for dietary plausibility by performing a regression of energy intake on body weight, but again no subject had a standardized residual greater than three SD above or below the mean, and therefore no subject was excluded for implausible energy intake. The Gibney et al. eating occasion definition was used to quantify eating frequency and each EO was defined as ≥50 cal and ≥15 min from any previous EO [9].

### Physical activity

Physical activity was measured by accelerometers (wGT3X-BT, Actigraph, LLC., Pensacola, FL). Physical activity was measured for seven days and on the same week as the in-person visit and dietary recalls. All accelerometer data was immediately downloaded and wear time was assessed. Days with less than 8 h of wear data were not considered acceptable, and only participants with ≥3 days of acceptable accelerometry data were included in the physical activity analysis. Subjects with valid data (*n* = 96) wore the accelerometers for a mean of 12.9 ± 1.6 h/day for 6.2 ± 1.5 days. Data from all acceptable days was averaged and included the following variables: minutes and percent time spent in light physical activity (LPA), moderate to vigorous PA (MVPA), and sedentary behavior (SED). Freedson adult cut offs were used to quantify and classify the accelorometry data [32].

### Statistics

Data was examined for normality, and transformations were made if the data was found to be significantly different from normal. The following outcome variables were non-normally distributed and were either log or inversely transformed before the analysis: BMI, VAT, SAT, hepatic fat, mean energy intake, percent dietary protein, saturated fat, dietary fiber, total fiber, soluble fiber, and insoluble fiber. However, non-transformed values are presented in the tables and figures for ease of interpretation. Chi-square, t-test, and MANCOVA analyses were used to assess differences in demographics, dietary intake variables, and adiposity and physical activity measures between the two eating frequency groups. In all models the following a priori covariates were included: sex, age and MVPA (when adiposity measures were the dependent variables). The interaction effect of eating frequency and will be tested in all models, and if significant the models will be split by sex to explore differences in the dependent variables. All analyses were performed by using SPSS version 20.0 (SPSS, Chicago, IL), and the significance was set at *p* ≤ 0.05. A post-hoc power analysis set at a type 1 error of 0.05 and a power of 80%, utilizing the means and standard deviations from the current data set revealed a medium effect size of 0.46 for SAT, 0.49 for BMI, 0.50 for VAT, and 0.64 for energy intake.

## Results

The basic demographic data and adiposity measures are presented in Table 1. Ninety-two subjects had complete anthropometric, dietary, and body composition data. The sample was 51% female and averaged 18.8 years of age.

**Table 1:** Subject Characteristics^a,b^

Physical and Adiposity Measures (*n* = 92***)***	All Subjects (92)	Males (45)	Females (47)
Age (y)	18.8 ± 0.4	18.7 ± 0.4	18.9 ± 0.4
Height (cm)	167.4 ± 9.8	188.3 ± 5.6	160.3 ± 7.5
Weight (kg)	67.1 ± 13.8	75.3 ± 13.3	59.2 ± 9.5
Waist circumference (cm)	84.6 ± 9.7	85.9 ± 10.6	83.4 ± 8.6
BMI (kg/m^2^)	23.8 ± 3.9	24.6 ± 4.1	23.1 ± 3.7
BMI percentile	59.9 ± 27.3	62.7 ± 28.6	57.2 ± 26.1
BMI z score	0.3 ± 1.0	0.4 ± 110	0.2 ± 0.9
Overweight Prevalence	21 (22.8)	11 (24.4)	10 (21.2)
Obese Prevalence	8 (8.7)	6 (13.3)	2 (4.3)
Overweight/Obese Prevalence	29 (31.5)	17 (37.8)	12 (25.5)
Total lean tissue (%)	73.6 ± 9.9	78.9 ± 9.1	68.5 ± 7.8
Total body fat (%)	26.4 ± 9.9	21.1 ± 9.1	31.5 ± 7.8
VAT (ml)	267.0 ± 124.4	286.9 ± 114.4	247.8 ± 131.8
SAT (ml)	999.7 ± 654.9	1020.8 ± 749.0	979.5 ± 557.6
Hepatic fat (ml)	29.2 ± 36.0	35.1 ± 44.9	23.5 ± 23.8
NAFLD	20 (21.7)	11 (24.4)	9 (19.1)

^a^Data presented as mean ±SD or n (%)

^b^NAFLD = Non Alcoholic Fatty Liver Disease, SAT = Subcutaneous Adipose Tissue, VAT = Visceral Adipose Tissue

Table 2 presents dietary and physical activity data. The average number of EOs per 24 h was 3.6, the average energy intake was close to 2000 kcals per day, and subjects averaged more than 60 min per day of MVPA.

**Table 2:** Dietary and Physical Activity Variables^a,b^

	All Subjects	Males (45)	Females (47)
Eating occasions per day	3.6 ± 1.1	3.4 ± 1.2	3.6 ± 1.1
Energy per eating occasion (kcal)	580.9 ± 241.6	715.3 ± 253.1	452.1 ± 139.9
Energy (kcal/d)	1941.9 ± 729.4	2345.8 ± 774.5	1555.2 ± 407.9
Total fat (g/day)	75.8 ± 31.5	92.6 ± 31.4	59.8 ± 22.0
Total protein (g/day)	82.7 ± 42.1	100.7 ± 50.8	65.5 ± 20.9
Total carbohydrate (g/day)	237.4 ± 94.1	281.9 ± 104.2	194.8 ± 57.7
Total saturated fat (g/day)	23.7 ± 11.4	29.1 ± 12.2	18.7 ± 7.8
Total sugars (g/day)	95.3 ± 52.6	107.2 ± 63.8	83.9 ± 36.2
Added sugars (g/day)	62.3 ± 46.0	79.2 ± 55.2	57.9 ± 32.3
Dietary fiber (g/day)	16.9 ± 7.5	18.9 ± 8.4	14.9 ± 6.1
Insoluble fiber (g/day)	11.3 ± 5.5	12.7 ± 6.2	10.0 ± 4.4
Soluble fiber (g/day)	5.4 ± 2.3	6.1 ± 2.4	4.8 ± 2.0
Min per day in MVPA	67.2 ± 27.0	69.6 ± 29.4	65.0 ± 24.6
Percent wear time in MVPA (%)	8.7 ± 3.4	8.9 ± 3.8	8.5 ± 3.1
Min per day in LPA	100.5 ± 32.4	102.6 ± 36.7	98.7 ± 28.0
Percent wear time in LPA (%)	12.9 ± 3.5	13.0 ± 4.0	12.8 ± 3.0
Min per day in SED	606.8 ± 81.4	610.0 ± 89.2	603.7 ± 73.9
Percent wear time in SED (%)	78.4 ± 4.7	78.1 ± 5.4	78.7 ± 4.2

^a^Data presented as mean ±SD

^b^*LPA* Light Physical Activity, *MVPA* Moderate to Vigorous Physical Activity, *SED* Sedentary Behavior, *Min* Minutes

Table 3 presents adiposity measures by the two eating frequency groups. Infrequent eaters compared to frequent eaters were slightly older (18.9 ± 0.4 vs. 18.6 ± 0.4 kg/m^2^, *p* = 0.05) and no significant difference in sex was found between eating frequency groups. Using MANCOVA analyses, infrequent eaters had 8% higher BMIs (24.8 ± 4.4 vs. 22.9 ± 3.2 kg/m^2^, *p* = 0.02)*,* 60% higher BMI z-scores (0.5 ± 1.0 vs. 0.2 ± 1.0, *p* = 0.03)*,* 21% higher VAT (298.3 ± 153.8 vs. 236.8 ± 78.2 ml, *p* = 0.03), 26% higher SAT (1150.1 ± 765.4 vs. 855.6 ± 494.6 ml, *p* = 0.03), and 8% higher total body fat (27.6 ± 10.8 vs. 25.3 ± 8.8%, *p* = 0.04) compared to frequent eaters. There was no significance difference in liver fat or prevalence of NAFLD between eating frequency groups. There was also no difference in physical activity variables between infrequent and frequent eaters. The interaction effect for eating frequency and sex was a trend or significant for BMI (*p* = 0.05), total body fat *(p* ≤ 0.01), total lean (*p* ≤ 0.01), VAT (*p* = 0.07), hepatic fat (*p* = 0.06). When stratifying the sample by sex there was a trend for male infrequent eaters to have 6% higher BMIs (25.3 ± 4.5 vs. 23.8 ± 3.5 kg/m^2^, *p* = 0.10) compared to male frequent eaters. Female infrequent eaters compared to female frequent eaters had 8% higher BMIs (24.2 ± 4.3 vs. 22.2 ± 2.8 kg/m^2^, *p* = 0.04), a trend for 5% lower lean tissue and a trend for (66.5 ± 8.3 vs. 70.2 ± 7.1%, *p* = 0.08) and a trend for 11% higher total body fat (33.5 ± 8.3 vs. 29.8 ± 7.1%, *p* = 0.08).

**Table 3:** Adiposity measures and metabolic parameters between eating frequency groups^a,b^

	All Infrequent Eaters (*n* = 45)	All Frequent Eaters (*n* = 47)	*p* value	Male Infrequent Eaters (*n* = 24)	Male Frequent Eaters (*n* = 21)	*p* value	Female Infrequent Eaters (*n* = 21)	Female Frequent Eaters (*n* = 26)	*p* value
Sex M/F^c^	24/21	21/26	0.53	–	–	–	–	–	–
Age (y)^c^	18.9 ± 0.4	18.7 ± 0.4	0.05	18.8 ± 0.4	18.6 ± 0.4	0.21	19.0 ± 0.4	18.8 ± 0.4	0.09
Height (cm)^c^	167.0 ± 10.4	167.8 ± 9.4	0.16	174.3 ± 5.7	175.5 ± 5.5	0.48	158.7 ± 8.1	161.5 ± 6.9	0.20
Weight (kg)^c^	69.4 ± 16.2	64.7 ± 11.2	0.29	77.1 ± 15.7	73.2 ± 9.8	0.33	61.0 ± 12.1	57.7 ± 6.6	0.24
Waist circumference (cm)^d^	86.2 ± 11.6	83.1 ± 7.2	0.19	87.8 ± 11.9	83.8 ± 8.7	0.15	84.4 ± 11.3	82.6 ± 5.8	0.19
BMI (kg/m^2^)^d^	24.8 ± 4.4	22.9 ± 3.2	0.02	25.3 ± 4.5	23.8 ± 3.5	0.10	24.2 ± 4.3	22.2 ± 2.8	0.04
BMI percentile^d^	64.0 ± 27.8	55.9 ± 26.6	0.08	65.5 ± 28.5	59.5 ± 29.2	0.22	62.4 ± 27.7	53.1 ± 24.4	0.17
BMI z score^d^	0.5 ± 1.0	0.2 ± 1.0	0.03	0.6 ± 1.1	0.3 ± 1.1	0.13	0.4 ± 0.9	0.1 ± 0.8	0.11
VAT (ml)^d^	298.3 ± 153.8	236.8 ± 78.2	0.03	307.9 ± 130.6	262.8 ± 89.6	0.12	287.5 ± 179.5	215.8 ± 61.6	0.18
SAT (ml)^d^	1150.1 ± 765.4	855.6 ± 494.6	0.03	1152.4 ± 856.0	870.3 ± 589.0	0.17	1147.7 ± 667.9	843.7 ± 415.1	0.06
Hepatic Fat (ml)^d^	31.4 ± 39.9	27.1 ± 32.1	0.35	34.7 ± 44.7	35.6 ± 46.2	0.90	27.6 ± 34.3	20.2 ± 8.7	0.26
NAFLD n (%)^c^	9 (20.0)	11 (23.4)	0.80	4 (16.7)	7 (33.3)	0.30	5 (23.8)	4 (15.4)	0.49
Total lean tissue (%)^d^	72.4 ± 10.8	74.7 ± 8.8	0.04	77.6 ± 10.2	80.3 ± 7.6	0.21	66.5 ± 8.3	70.2 ± 7.1	0.08
Total body fat (%)^d^	27.6 ± 10.8	25.3 ± 8.8	0.04	22.4 ± 10.2	19.7 ± 7.6	0.21	33.5 ± 8.3	29.8 ± 7.1	0.08

^a^Data presented as mean ± SD or n (%)

^b^*NAFLD* Non Alcoholic Fatty Liver Disease, *SAT* Subcutaneous Adipose Tissue, *VAT* Visceral Adipose Tissue

^c^A t-test (for continuous variables) and chi-square analysis (for categorical variables) assessed differences in sex, age, height, and weight between groups

^d^MANCOVA analysis of adiposity measures between Infrequent Eaters and Frequent Eaters (*n* = 92). For the combined sample a priori covariates included: age, sex, and percent time spent in moderate to vigorous physical activity. When the sample was split by sex a priori covariates included: age, sex, and percent time spent in moderate to vigorous physical activity

Dietary and physical activity variables between frequent eaters and infrequent eaters are depicted in Table 4. T-tests found that infrequent eaters compared to frequent eaters ate 44% less often (2.5 ± 0.2 vs. 4.5 ± 0.8, *p* ≤ 0.01) and ate 27% more calories per EO (670.9 ± 254.2 vs. 494.6 ± 195.1 kcals, *p* ≤ 0.01). Infrequent eaters compared to frequent eaters consumed 21% less daily energy intake, or on average 445 fewer calories per day (1714.8 ± 542.7 vs. 2159.5 ± 819.7 kcals, *p* ≤ 0.01). Infrequent eaters also consumed significantly less protein (71.8 ± 25.9 vs. 93.2 ± 51.4 g/day, *p* ≤ 0.01), fat (70.0 ± 26.4 vs. 81.4 ± 35.0 g/day, *p* = 0.01), carbohydrate (202.2 ± 69.4 vs. 271.1 ± 102.6 g/day, *p* ≤ 0.01), saturated fat (21.1 ± 8.7 vs. 26.1 ± 13.1 g/day, *p* = 0.02), total sugar (77.8 ± 35.0 vs. 112.1 ± 60.9 g/day, *p* ≤ 0.01), total fiber (13.5 ± 5.7 vs. 20.0 ± 7.8 g/day, *p* ≤ 0.01), insoluble fiber (9.1 ± 4.3 vs. 13.5 ± 5.7 g/day, *p* ≤ 0.01), and soluble fiber (4.4 ± 1.6 vs. 6.4 ± 2.5 g/day,*p* ≤ 0.01). The interaction effect for eating frequency and sex was significant for all dietary variables, but not significant for any physical activity measures. When examining sex differences, all of the above dietary findings remained significant or trending toward significance between male infrequent and frequent eaters, while there were no significant differences in dietary fat, protein, or saturated fat between female infrequent and female frequent eaters.

**Table 4:** Dietary intake and physical activity between eating frequency groups^a,b^

	All Infrequent Eaters (*n* = 45)	All Frequent Eaters (*n* = 47)	*p* value	Male Infrequent Eaters (*n* = 24)	Male Frequent Eaters (*n* = 21)	*p* value	Female Infrequent Eaters (*n* = 21)	Female Frequent Eaters (*n* = 26)	*p* value
Eating occasions per day^c^	2.5 ± 0.2	4.5 ± 0.8	≤0.01	2.5 ± 0.2	4.4 ± 0.9	≤0.01	2.5 ± 0.2	4.5 ± 0.7	≤0.01
Energy per eating occasion (kcal)^c^	670.9 ± 254.2	494.6 ± 195.1	≤0.01	781.9 ± 286.2	639.2 ± 187.9	0.06	544.2 ± 127.6	377.8 ± 100.5	≤0.01
Energy (kcal/day) ^c^	1714.8 ± 542.7	2159.5 ± 819.7	≤0.01	1992 ± 561.4	2750.1 ± 797.1	≤0.01	1397.6 ± 296.0	1682.5 ± 445.4	0.02
Total fat (g/day) ^d^	70.0 ± 26.4	81.4 ± 35.0	0.04	81.5 ± 28.1	105.2 ± 30.9	0.02	56.7 ± 17.1	62.2 ± 25.3	0.85
Total protein (g/day) ^d^	71.8 ± 25.9	93.2 ± 51.4	≤0.01	81.0 ± 27.4	105.2 ± 30.9	≤0.01	61.3 ± 19.8	65.6 ± 20.9	0.15
Total carbohydrates (g/day) ^d^	202.2 ± 69.4	271.1 ± 102.6	≤0.01	236.0 ± 67.1	334.3 ± 115.3	≤0.01	163.5 ± 49.6	220.0 ± 51.6	≤0.01
Total saturated fat (g/day) ^d^	21.1 ± 8.7	26.1 ± 13.1	0.02	24.8 ± 9.3	33.9 ± 13.4	0.02	17.1 ± 5.7	19.9 ± 9.0	0.64
Total sugars (g/day) ^d^	77.8 ± 35.0	112.1 ± 60.9	≤0.01	88.2 ± 37.3	128.9 ± 80.3	0.10	65.9 ± 28.8	98.5 ± 35.4	≤0.01
Added sugars (g/day) ^d^	60.7 ± 36.5	75.6 ± 52.9	0.14	70.6 ± 39.3	89.0 ± 68.9	0.75	49.4 ± 30.1	64.7 ± 33.0	0.14
Dietary fiber (g/day) ^d^	13.5 ± 5.7	20.0 ± 7.8	≤0.01	15.4 ± 5.9	23.0 ± 9.1	≤0.01	11.4 ± 4.7	17.7 ± 5.7	0.02
Insoluble fiber (g/day) ^d^	9.1 ± 4.3	13.5 ± 5.7	≤0.01	10.2 ± 4.6	15.6 ± 6.7	≤0.01	7.8 ± 3.7	11.8 ± 4.1	0.04
Soluble fiber (g/day) ^d^	4.4 ± 1.6	6.4 ± 2.5	≤0.01	5.2 ± 1.5	7.2 ± 2.8	≤0.01	3.6 ± 1.3	5.7 ± 2.0	0.02
Min per day in MVPA ^e^	63.5 ± 27.0	70.8 ± 26.8	0.29	68.0 ± 31.7	71.4 ± 27.2	0.72	58.5 ± 20.0	70.2 ± 27.0	0.15
Percent wear time in MVPA (%)^e^	8.3 ± 3.7	9.0 ± 3.1	0.50	9.0 ± 4.6	8.9 ± 2.6	0.95	7.7 ± 2.4	9.1 ± 3.5	0.17
Min per day in LPA^e^	96.8 ± 32.0	104.2 ± 32.7	0.16	94.4 ± 37.2	111.9 ± 34.5	0.16	99.5 ± 25.6	98.0 ± 30.3	0.75
Percent wear time in LPA (%)^e^	12.5 ± 3.5	13.3 ± 3.5	0.20	12.0 ± 3.9	14.1 ± 4.0	0.13	13.1 ± 3.0	12.5 ± 3.1	0.94
Min per day in SED^e^	607.1 ± 95.8	606.5 ± 65.7	0.93	612.0 ± 108.0	607.8 ± 63.9	0.96	607.1 ± 95.8	606.5 ± 65.7	0.85
Percent wear time in SED (%)^e^	79.1 ± 4.8	77.7 ± 4.7	0.15	79.0 ± 5.6	77.0 ± 5.0	0.28	79.2 ± 3.8	78.3 ± 4.5	0.34

^a^Data presented as mean ± SD

^b^LPA = Light Physical Activity, MVPA = Moderate to Vigorous Physical Activity, SED = Sedentary Behavior, Min = Minutes

^c^T-test assessed differences in eating occasions per day, energy intake, and energy intake per eating occasion between groups

^d^MANCOVA analysis of dietary variables between Infrequent Eaters and Frequent Eaters. A priori covariates used for the combined data set were age and sex. The a priori covariate for males and females was age

^e^MANCOVA analysis of physical activity variables between Infrequent Eaters and Frequent Eaters. A priori covariates used for the combined data set were age and sex. When the sample was split by sex the a priori covariate was age

## Discussion

To our knowledge, this is the first analysis to examine the relationship between eating frequency and dietary and adiposity measures in a sample of exclusively Hispanic college freshmen. To date, the dietary habits and obesity risk of this population remains almost completely unstudied. In the present analysis, infrequent eaters consumed significantly fewer calories per day, yet had significantly higher BMI, BMI z-scores, body fat percentage, and visceral and subcutaneous adipose tissue, while showing no significant differences in physical activity measures. These adiposity findings among infrequent and frequent eaters seemed to driven by females more so than males, but both sexes showed trends in the same direction for all adiposity outcomes. These findings are consistent with other retrospective analyses [12–15] that have shown a positive association between eating frequency and caloric intake while showing an inverse association with adiposity measures.

To date, the majority of epidemiology studies have shown an inverse association between eating frequency and adiposity [13–19], while a few studies have found no relationship or an inverse association [20–22]. A longitudinal study by Ritchie et al. [15], with 2372 African American and Caucasian girls (9–19 y), found that lower meal frequency was related to greater increases in BMI and waist circumference over a ten year period, independent of socioeconomic status, total energy intake, and physical activity levels. Other studies have found increased eating frequency to be inversely related to waist circumferences [15, 17, 33] and body fat percent as measured by skinfolds [12]. Increased body fat percentages, regardless of BMI levels, have also been related to increased inflammation and cardiometabolic risk factors [34] and for every kilogram increase in bodyweight the risk of type 2 diabetes increases by 5.4% [35]. Furthermore, 43% of female infrequent eaters were at or above the 35% body fat percentage cut off for obesity compared to only 30% of frequent eaters. Similarly, 38% of male infrequent eaters were at or above the 25% body fat percentage cut off for obesity compared to only 19% of frequent eaters [36, 37]. As a group, the infrequent eaters were also 0.2 kg/m^2^ from the overweight cutoff, thus as this population ages the difference in adiposity measures between infrequent and frequent eaters may become even more apparent. A recent meta-analysis by Schoenfeld et al. including 15 randomized controlled trials addressing the effects of eating frequency on changes in weight and body composition found that increases in eating frequency were associated with reductions in fat mass and body fat percentage, as well as increases in fat-free mass. However, these findings need to be interpreted cautiously as they seem to be driven by a single study [38]. The above meta-analysis was comprised of an exclusively adult population and to our knowledge, there has yet to be a randomized controlled trial investigating the potential association between eating frequency and obesity risk in any youth population, let alone one of Hispanic descent.

Interestingly, this population of Hispanic College Students had a lower percentage of overweight and obesity risk compared to national averages [39]. Ninety four percent of subjects met or exceeded the physical activity guidelines for Americans of 150 min of moderate to vigorous physical activity per week [40]. However, diet quality in this population was lacking. While, infrequent eaters consumed significantly less fiber (6.5 g/day) than frequent eaters, only five subjects (four females and one male) within the entire sample met the Recommended Daily Allowance (RDA) for fiber [41]. Furthermore, only 39% of the sample met the recommendation for less than 10% of their calories from added sugar and only 43% of the sample met the RDA for less than 10% of their calories from saturated fat [41]. Also, 18% of the sample (8 males and 9 females) did not meet the RDA for protein of 0.8 g/kg. Thus, more research is needed to further explore the dietary habits of Hispanic college freshmen and if there is any potential relationship between eating frequency and diet quality.

There are numerous potential mechanisms to explain our findings that infrequent eaters consumed significantly fewer calories per day, yet had significantly higher adiposity measures than frequent eaters. The first being that increased eating frequency has consistently been related to increased satiety measures in adult populations [42–45]. Smeets et al. [43] found that consuming 3 EOs per day compared 2 EOs per day resulted in greater satiety in a sample of 14 females (19–29 y). However, in a cross-over controlled feeding study by Leidy et al. [42] with 13 overweight or obese males, less frequent eating (3 EOs) vs. frequent eating (6 EOs) led to higher satiety throughout the day, but no difference in ghrelin or peptide YY was observed between groups. These studies controlled for calories and so did not address the impact eating frequency may have on ad libitum food intake. Thus, Speechly et al. [44] conducted a cross-over study with eight lean males (19–29 y) where an isocaloric breakfast was consumed in one EOs or five separate EOs consumed every hour over the testing period. Subjects who consumed breakfast in one EO ate 27% more at a subsequent ad libitum lunch, highlighting that infrequent eating may lead to poorer appetite control. Interestingly, utilizing an identical study design with seven obese men (20–55 y), Speechly et al. [46] replicated the findings and found the exact same increase (27%) in the ad libitum lunch intake after the single meal. It is reasonable to infer that increased satiety observed in regards to increased eating frequency may reduce the motivation to eat and therefore reduce energy intake overall. However, the vast majority of the eating frequency research, including this study shows a positive relationship between eating frequency and energy intake, which very well could be an artifact of under-reporting which is discussed below. It is also plausible that reduced eating frequency may result in eating behaviors that resemble binge eating which has been related to increases in metabolic disease parameters and adiposity measures [47]. Thus, much more research is needed to examine the exact mechanisms of how eating patterns impact satiety, hunger, and ad libitum dietary intake in free-living populations.

Another potential mechanism to consider is how eating frequency impacts metabolic rates. Popular media have consistently advocated more frequent eating or grazing as a healthy habit that may “stoke” or “rev” one’s metabolism. A recent review of popular media sources found the ideal eating frequency recommendation given was 6 EOs per day [48]. Yet, not a single study to date has found a statistically significant difference in total energy expenditure between 1, 2, 3, 5, 6 or even 7 EOs in a 24-h period [43, 49–52]. A cross-over study with eight young adult males (18–23 y) examined the difference in metabolic rate between 2 EOs and 6 EOs per day, which were isocaloric, subjects stayed on each dietary regimen for two weeks and occupied a whole room calorimeter for two 31-h periods [49]. This study found no differences in metabolic rate or energy expenditure between the two EO conditions, despite a small, albeit significant, observed weight gain in the 2 EOs per day condition. However, this study kept the activity patterns constant and eating frequency may subsequently increase physical activity levels, however this and previous analyses in Hispanic youth have not found a difference in physical activity measures between eating frequency groups. In a similar two day cross-over study with 13 male and female young adults (18–23 y), no significant differences in 24-h energy expenditure as measured by a whole room calorimeter were found between 2 EOs and 7 EOs per day conditions [50]. Thus, to date it does not appear that eating more frequently increases metabolic rates and given the body of research the likelihood that this would have led to the current findings is quite low. Yet, given the pervasiveness of the current media recommendation to increase eating frequency to increase metabolic rate further research into the potential impacts of eating frequency on obesity and metabolic disease risk is warranted to better inform the general public of the potential benefits of frequent eating or deleterious outcomes of infrequent eating.

Another potential mechanism to consider when investigating eating frequency is the potential impact on the thermic effect of food (TEF). TEF is defined as the increase in metabolic rate after the ingestion of a meal. To date, studies examining the impact of eating frequency on TEF have yielded mixed results. One study found an increase in TEF in 1 EO compared to 6 EOs [53], while another study showed an increase in TEF in 4 EOs compared to 1 EO [54], whereas the majority of studies show no significant effect of eating frequency on TEF [43, 55, 56]. Tai et al. [53] examined the effect of 1 large EO of 750 cal vs. 6 smaller EOs of 125 cal provided every 30 min over the same period on TEF in seven women (23–30 y) and found that the one large EO resulted in a slightly higher TEF of 3.4%. The authors hypothesized that this was due to a more rapid absorption of nutrients given that gastric emptying is slower when food is given continuously [57]. Similarly, another study by Leblanc et al. in six subjects (21–28 y) compared 1 vs. 4 EOs given over a four-hour period and found that the 4 EO pattern resulted in a slightly higher TEF. However, eating one time per day is not reflective of normal eating patterns seen in free-living populations and in the current analysis there was not a single subject who averaged 1 EO per day. Furthermore, a comprehensive review by Bellisle et al. [56] concluded that there is no strong evidence to support a biologically significant difference in TEF in response to different eating frequency, and furthermore, the role of TEF on body weight regulation itself remains controversial [58]. To our knowledge, no research study has investigated any potential mediating effect that an increase in TEF from an increase or decrease in eating frequency or a change in macronutrient consumption due to changes in eating frequency may have on adiposity parameters. In addition, no study has examined how eating frequency impacts TEF in a youth population, yet given the body of research the likelihood that this would have led to the current findings is also quite low.

Another possible mechanism involves lipid metabolism. Previous studies have found infrequent eating to be associated with higher circulating triglycerides [13, 14]. Infrequent eaters also consistently show an increased caloric intake per EO, and binge eating behaviors have been previously linked to increased triglycerides [14, 47]. Infrequent eaters have also shown increased visceral adipose tissue [14] and the accumulation of visceral adipose tissue has been positively associated with fasting insulin and triglycerides [14, 59]. It is also hypothesized that visceral fat increases hepatic portal free fatty acid concentrations, which in turn are stored as triglycerides, stimulate hepatic gluconeogenesis, and hinder hepatic clearance of insulin, thus promoting a vicious cycle of hyperinsulinemia, elevated plasma glucose concentrations, and dyslipidemia [60]. Furthermore, increased risk of NAFLD has been found to be associated with dyslipidemia [61]. It has also been hypothesized that increased triglycerides and free fatty acids from VAT are the first hit in the progression of NAFLD, however we did not see any significant difference in hepatic fat by eating frequency groups, yet 20% of this highly active young adult Hispanic population did meet the diagnostic criteria for NAFLD [61]. The current analysis is in agreement with previous finding that infrequent eaters have higher visceral adipose tissue than frequent eaters and thus more research, especially randomized controlled feeding trials analyzing the possible causal relationship between visceral adiposity, triglycerides, eating frequency, NAFLD, and metabolic disease risk is merited.

Another potential explanation for the current findings is that healthy behaviors tend to cluster together [62]. In the current study, frequent eaters are just consuming more in general - more calories, total carbohydrates, total protein, total fat, total fiber, and total sugars. Conversely, infrequent eaters may be more apt to partake in other unhealthy behaviors. Infrequent eaters did consume less fiber per day compared to frequent eaters, but only five subjects met the recommendation for fiber intake. Previous research has also found that the differences in adiposity measures by eating frequency groups were mediated by differences in physical activity [63]. However, no significant differences in physical activity were seen between eating frequency groups within this analysis, and these subjects were highly active with the vast majority meeting the physical activity guidelines for Americans. Thus, further investigation is needed into understand if or how eating frequency may influence other behaviors.

An important confounder in the current analysis is a potential higher prevalence of under-reporting of intake and potentially even omitting entire eating occasions by overweight/obese subjects which has been seen previously in eating frequency research. Previous research has highlighted the possible role of dietary underreporting [64], particularly in an overweight/obese samples. Additionally, per the USDA an active 18–19 year old male would need 3000–3200 kcal per day and an active 18–19 year old female would need 2200–2400 kcal per day to maintain weight and only nine males and five females met or exceeded these numbers [65], thus some degree of underreporting within this sample is likely. Yet, within the current analysis we took multiple steps to assess energy intake plausibility, however we cannot be certain under-reporting was not a confounder in the current findings.

There are a several potential limitations in the current analysis. The first is that this is a cross-sectional study and thus causation cannot be assessed. Additionally, this analysis included normal, overweight, and obese subjects. However, we chose to include all weight categories given that no other group has investigated the dietary habits and obesity risk of Hispanic college freshmen. Another limitation is that this sample may not be representative of other Hispanic college populations, as only 28% of our sample was overweight or obese, which is lower than national prevalence rates for this age range and ethnicity/race. In addition, this university has consistently been ranked in national polls as one of the healthiest colleges in the nation [66, 67] and the current population was extremely active, on average participating in more than 60 min in MVPA per day. Another potential limitation is that we were sufficiently powered to examine differences in adiposity and diet between eating frequency groups, however, we were not prospectively powered for splitting the sample by sex and the observed power in these sub analyses was markedly less, especially for adiposity measures. But regardless, increased eating frequency was linked to reduced adiposity, even within this fairly healthy population. Thus, the current findings may not be universal among all Hispanic youth populations, although previous work has found similar relationships in younger overweight and obese minority youth [13, 14].

## Conclusion

In summary, eating less than three eating occasions per day is associated with increased BMI, BMI z-scores, body fat percentage, visceral and subcutaneous adipose tissue, despite being linked to decreased daily energy intake in a population of Hispanic college freshmen. Given that the first year of college is such a pivotal time in the development of lifelong habits it is important to identify nutrition behaviors that may potentially reduce the risk of obesity later in life. These results as well as previous findings support that further longitudinal trials are needed to investigate the potential causal relationship between eating frequency and obesity risk in Hispanic youth, as well as establish the need for intervention work in this area.

## Abbreviations

BMI: Body Mass Index
EO: Eating Occasion
FoV: Field of View
GRAPPA: GeneRalized Auto-calibrating Partially Parallel Acquisitions
LPA: Light Physical Activity
MRI: Magnetic Resonance Imaging
MVPA: Moderate to Vigorous Physical Activity
NAFLD: Non-Alcoholic Fatty Liver Disease
NDS-R: Nutrition Data System for Research
ROI: Region of Interest
SAT: Subcutaneous Adipose Tissue
SED: Sedentary Behavior
TE: Echo Time
TR: Repetition Time
UT-Austin: University of Texas at Austin
VAT: Visceral Adipose Tissue
